# Activity, polypeptide and gene identification of thylakoid Ndh complex in trees: potential physiological relevance of fluorescence assays

**DOI:** 10.1111/j.1399-3054.2012.01598.x

**Published:** 2012-09

**Authors:** Patricia H Serrot, Bartolomé Sabater, Mercedes Martín

**Affiliations:** Departamento de Biología Vegetal, Universidad de AlcaláAlcalá de Henares, 28871 Madrid, Spain

## Abstract

Three evergreen (*Laurus nobilis*, *Viburnum tinus* and *Thuja plicata*) and two autumnal abscission deciduous trees (*Cydonia oblonga* and *Prunus domestica*) have been investigated for the presence (zymogram and immunodetection) and functionality (post-illumination chlorophyll fluorescence) of the thylakoid Ndh complex. The presence of encoding *ndh* genes has also been investigated in *T. plicata*. Western assays allowed tentative identification of zymogram NADH dehydrogenase bands corresponding to the Ndh complex after native electrophoresis of solubilized fractions from *L. nobilis*, *V. tinus*, *C. oblonga* and *P. domestica* leaves, but not in those of *T. plicata*. However, Ndh subunits were detected after SDS-PAGE of thylakoid solubilized proteins of *T. plicata*. The leaves of the five plants showed the post-illumination chlorophyll fluorescence increase dependent on the presence of active Ndh complex. The fluorescence increase was higher in autumn in deciduous, but not in evergreen trees, which suggests that the thylakoid Ndh complex could be involved in autumnal leaf senescence. Two *ndhB* genes were sequenced from *T. plicata* that differ at the 350 bp 3′ end sequence. Comparison with the mRNA revealed that *ndhB* genes have a 707-bp type II intron between exons 1 (723 bp) and 2 (729 bp) and that the UCA 259th codon is edited to UUA in mRNA. Phylogenetically, the *ndhB* genes of *T. plicata* group close to those of *Metasequoia*, *Cryptomeria*, *Taxodium*, *Juniperus* and *Widdringtonia* in the cupresaceae branch and are 5′ end shortened by 18 codons with respect to that of angiosperms.

## Introduction

The plastid *ndh* genes encode components of the thylakoid Ndh complex, which is analogous to the NADH dehydrogenase or complex I (EC 1.6.5.3) of the mitochondrial respiratory chain and catalyzes the transfer of electrons from NADH to plastoquinone (Sazanov et al. 1998, Casano et al. 2000, Rumeau et al. 2005, Martín et al. 2009). In concerted action with electron-draining reactions, the Ndh complex protects against photo-oxidative-related stresses (Martín et al. 1996, Endo et al. 1999), probably by contributing to poising the redox level of the cyclic photosynthetic electron transporters (Casano et al. 2000, Joët et al. 2002, Martín et al. 2009). Alternatively, Yamamoto et al. (2011) have proposed that the Ndh complex transfers electrons from reduced ferredoxin to plastoquinone, providing a cyclic electron transport pathway additional to the commonly accepted model in which ferredoxin directly donates electrons to the PQ/*cyt.b*_6_*f* intermediary electron pool (Kurisu et al. 2003). By feeding excess electrons, the overexpression of the Ndh complex, combined with the low level of superoxide dismutase (Casano et al. 2000, Abarca et al. 2001a, 2001b), triggers the levels of reactive oxygen species and induces programmed leaf cell death (Zapata et al. 2005). Chloroplasts contain only one Ndh complex per 100 to 200 photosystems (Sazanov et al. 1998, Casano et al. 2000), which in addition to the difficulties involved in the proteomic handling of its highly hydrophobic subunits (Darie et al. 2005) and the instability of the purified preparation (Martín et al. 2009) constitutes the reason for which most investigations on the Ndh complex thus far have consisted mainly of genetic approaches and molecular characterizations in rapidly growing monocarpic plants such as pea, barley, maize, *Arabidopsis* and tobacco.

The higher sensitivity of *ndh* gene defective plants to stress and the consistent presence of the plastid *ndh* genes in most photosynthetic plants in the line leading from certain charophycean green algae to land plants suggest that the Ndh complex is necessary or provides advantages for photosynthesis in the highly fluctuating terrestrial environment (Martín and Sabater 2010). Accordingly, the Ndh complex could be involved in the photosynthetic adaptation of leaves to the rapid and extreme light and temperature variations to which many perennial plants are exposed. However, despite the frequently described presence of *ndh* genes, to our knowledge, no investigation has yet been published on the presence of the functional Ndh complex in trees. The difficulties involved both in selecting physiologically uniform leaves and in the comparison of assays along the successive seasons of the year pose serious challenges to the investigation of the functional role of the Ndh complex in trees. In order to establish easy and rapid tests for extensive investigation in perennial plants, the presence (by zymogram and immunodetection) and in situ functionality (by the increase of chlorophyll fluorescence after transition to minimum light) of the Ndh complex were investigated in two deciduous (*Cydonia oblonga* and *Prunus domestica*) and three non-deciduous (*Laurus nobilis*, *Thuja plicata* and *Viburnum tinus*) trees. To settle conflicting immunoassay results between native and SDS-PAGE electrophoresis obtained from *T. plicata*, complementary molecular biology investigations were carried out. Two complete *ndhB*, one complete *ndhC* gene and, partially, other *ndh* genes of *T. plicata* were sequenced. In contrast to evergreen trees, deciduous trees showed an increase of the autumnal activity of the Ndh complex (as estimated by chlorophyll fluorescence assays) prior to leaf senescence.

## Materials and methods

### Plant material

Fresh specimens of adult *C. oblonga*, *L. nobilis*, *P. domestica*, *T. plicata* and *V. tinus* were obtained from the fields close to the campus of the University of Alcalá. *Hordeum vulgare* cv. Aspen was grown in the growth chamber as described (Martín et al. 1996) and primary leaves of 14-day-old plants were used. *Nicotiana tabacum*, wt (cv. Petit Havana) and Δ*ndhF* (transgenic defective in the *ndhF* gene) were grown as described (Martín et al. 2004).

### Leaf protein crude extracts and thylakoid isolation

Whole-leaf extracts were obtained by homogenization of 0.5 g leaves with liquid nitrogen in a mortar with 2 ml of 50 m*M* potassium phosphate, pH 7.0, 1 m*M* L-ascorbic acid, 1 m*M* EDTA, 1% polyvinylpyrrolidone (PVP) and 2% Triton X-100. The suspensions were gently stirred for 30 min and then centrifuged at 20 000 *g* for 30 min. Thylakoid isolations were carried out as described (Martín et al. 2009). For SDS-PAGE, the thylakoid pellets were resuspended in 1–2 mL of the extraction buffer and SDS added to a final concentration of 1%. The suspension was gently stirred for 20 min at 4°C and centrifuged at 17 000 *g* for 5 min.

### Protein gel electrophoresis, zymograms and immunoassays

Native PAGE was carried out at 5°C in the presence of 0.1% Triton X-100 as described (Martín et al. 2009). NADH dehydrogenase zymograms were developed by incubating the gel for 20 to 30 min at 30°C in darkness with 50 m*M* potassium phosphate pH 8.0, 1 m*M* EDTA, 0.2 m*M* NADH and 0.5 mg ml^−1^nitroblue tetrazolium. In the control without NADH, no stain developed. The activity band corresponding to the Ndh complex was identified by immunoblotting (Casano et al. 2000, Martín et al. 2009).

For other immunoblot analyses, samples were subjected to SDS-PAGE and transferred to polyvinylidene difluoride membranes (Millipore, Bedford, MA). NDH-A, NDH-D, NDH-F and NDH-J polypeptides were revealed using antibodies described previously (Zapata et al. 2005, Martín et al. 2009). The different immunocomplexes were detected with the alkaline phosphatase western-blotting analysis system (Roche Mannheim, Germany).

### Chlorophyll fluorescence induction analysis

Assays were carried out in the field with intact attached fully expanded healthy leaves. Chlorophyll fluorescence changes were measured with an Opti-Sciences (ADC BioScientific Ltd., Hertfords, UK) OS1-FL modulated chlorophyll fluorometer. Leaves were dark-adapted with clips for 30 min after which they received 2 min minimum light (0.1 µmol photon m^−2^ s^−1^ PAR) followed by 5 min higher relative light (0.15 µmol photon m^−2^ s^−1^ PAR) and, again, 9 min of minimum light. 0.8 s saturating flashes (5000 µmol photon m^−2^ s^−1^ PAR) were applied at 1, 3, 4, 5 and 6 min of light incubation. Fluorescence was recorded every 0.1 s and collected data were represented using the Grafit, Erithacus software (Surrey, UK). Assays were repeated at least three times. The increase of fluorescence after relative high to minimum light transition is currently attributed to the reduction of plastoquinone mediated by the thylakoid Ndh complex in higher plants (Burrows et al. 1998, Kofer et al. 1998, Shikanai et al. 1998, Martín et al. 2004, 2009, Wang and Portis 2007) and by the nuclear encoded NAD(P)H dehydrogenase (Nda2) in algae lacking the Ndh complex (Desplasts et al. 2009).

### Isolation of DNA and RNA and reverse transcription of RNA

DNA was extracted from 0.5 g fresh or frozen specimens using the protocol described by Tel-Zur et al. (1999). Total RNA was isolated from 1 g fresh or frozen specimens with the Concert™ Plant RNA Reagent method and treated with RQ1 RNase-free DNase I (Promega, Madison, WI). RNA yields ranged between 0.2 and 0.4 mg g^−1^ plant tissue. Reverse transcription was performed with 5 µg total RNA and reverse transcriptase (Superscript™ II RT; Invitrogen GmbH, Karlsruhe, Germany) using random primers. DNA and specific transcript amplifications were performed using AccuPrime™*Taq* DNA Polymerase (Invitrogen GmbH). Polymerase chain reaction (PCR) mixtures were supplemented with 0.1% BSA (w/v) and 1% PVP (w/v) to release *Taq* DNA polymerase inhibitors present in nucleic acid preparations (Xin et al. 2003). Cycling conditions were one cycle at 94°C for 5 min and 35 cycles of 94°C for 60 s, 46°C for 60 s and 68°C for 120–180 s. After agarose gel electrophoresis and purification (QIAquick Gel Extraction Kit; Qiagen GmbH, Hilden, Germany) samples were sequenced on an Applied Biosystems automatic sequencer. The sequences of the amplified fragments were captured with online available Chromas programs and alignments were done by eye.

### List of primers

B_1_: CATAGATATAGTGATAATAAG; B_2_: GGGAATGTTTTTATGTGGTGCTAA; B_3_: CCTCATTAGACCGAAT ATCTC; B_4_: CCGTTACAGATAGAAGTGC; B_5_: CCATTT CATCAATGGACTCC; B_6_: GAAAGAAAAGCAACGACT GG; B_7_: GTAGCTGCTTCAGCTTTAG; B_8_: GGTCAAATTGGATATATCC; B_9_: GGATATGCAAGTGTGATAAC; B_10_: GGATTATGCAGGATTATATATG; B_11_: ATCCTGCATAATCTCGAATG; B_12_: GGAAAACTCTAT CTATTCTGGTG; B_13_: GATAGGACTCTTTATGAGTGC; B_14_: GTTATCTTCTTCAACC; B_15_: AAGATCCCCTTTAAGA; B_16_: TTTTTTTTTTTGTTTGTTGTGGGGGGTGT; B_17_: TTTTTGTTTGTTGTGGG; B_18_: AAGGGTATCCTGAGCAATCG; B_19_; CGGAACAGATCTACTAATTC; B_20_; CTGAATCCTCTTCCTTCATAC; F2: CCTCTTCACGTATGGTTACC; F4: ACCAGAAGCAAGCAAGAGGT.

B_1_–B_20_ primers were used for amplifications of *ndhB* gene sequences. F_2_ and F_4_ primers were used for amplifications of *ndhF* gene sequences.

### Phylogenetic analyses

Phylogenetic trees using protein sequences were constructed with the EBI ClustalW server using the percentage identity (PID). Phylogenetic trees using DNA sequences were constructed in the NCBI server using the Fast Minimum Evolution method.

### Database accession numbers

Gene sequences first described in this paper were in the EMBL database with accession numbers: EF421240, EF421241, EF421243 and EF421244.

## Results

### Zymogram and immunoidentification of the Ndh complex

**Fig. 1:**
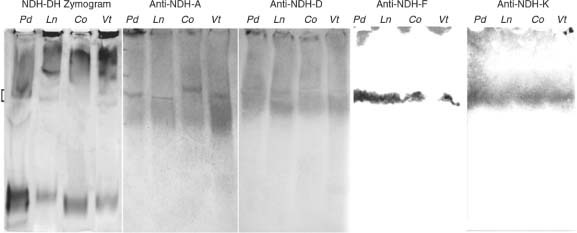
Zymogram of NADH dehydrogenase activities and immunoblot identification of the Ndh complex in four angiosperm trees. Solubilized thylakoid proteins from *Laurus nobilis* (*Ln*), *Cydonia oblonga* (*Co*), *Viburnum tinus* (*Vt*) and *Prunus domestica* (*Pd*) were separated by native electrophoresis and revealed for NADH dehydrogenase (NADH-DH) activity and, after membrane blotting, immunoassayed with antibodies against the NDH-A, NDH-D, NDH-F and NDH-K polypeptides. The whole gel and membrane photos are shown to assess the identification of the activities (marked with a bracket at left) corresponding to the Ndh complex as that containing protein detected with NDH antibodies.

The thylakoid Ndh complex can be tentatively identified by its NADH dehydrogenase activity and immunoreaction with antibodies against NDH polypeptides after native electrophoresis (Casano et al. 2000, Martín et al. 2004, 2009). Accordingly, extracts of proteins solubilized from thylakoids with 2% Triton X-100 were subjected to native electrophoresis and tested for the presence of NADH dehydrogenase activity by reduction of nitroblue tetrazolium with NADH. Zymograms showed several activity bands in the extracts of *L. nobilis*, *C. oblonga*, *V. tinus* and *P. domestica* thylakoids (Fig. 1). The most intense bands probably correspond to diaphorases, which also have NADH dehydrogenase activity. Probably, only one, low activity band in each plant (almost undetectable in the *C. oblonga* lane) corresponds to the thylakoid Ndh complex because, after subsequent transfer, antibodies raised against the NDH-A subunit of the barley Ndh complex recognized a closely migrating band. With variable band sharpness, similar results were obtained with antibodies against the NDH-F and NDH-K subunits. Antibodies raised against NDH-D detected several additional bands migrating in the vicinity of those detected by NDH-A that could be attributed to inactivated forms of the Ndh complex retaining a reactive NDH-D subunit (Fig. 1). The electrophoretic mobility of the Ndh complex differs slightly among the four plants (approximately 25% of the front) and is indicated by the bracket on the left side of Fig. 1.

**Fig. 2:**
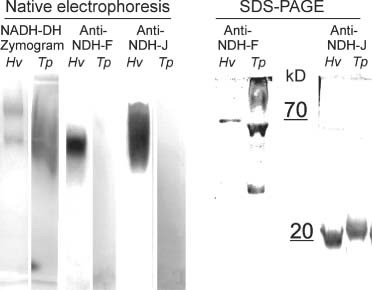
Native electrophoresis, SDS-PAGE and immunoblot related to the Ndh complex of *Thuja plicata*. Left side: zymograms revealing NADH dehydrogenase (NADH-DH) activities after native electrophoresis of *Hordeum vulgare* (*Hv*) and *Thuja plicata* (*Tp*) thylakoid extracts and, after membrane blotting, immunoassays with antibodies against NDH-F and NDH-J polypeptides. Right side: immunoassays with antibodies against NDH-F and NDH-J polypeptides of thylakoid extracts of *H. vulgare* and *T. plicata* separated by SDS-PAGE. The central lane indicates the migration of 70 and 20 kDa markers.

Native electrophoresis and zymograms also detected NADH dehydrogenase activities in the Triton X-100 extract from *T. plicata* thylakoids (left side of Fig. 2). However, no activity could be attributed to the thylakoid Ndh complex because, as shown with NDH-F and NDH-J antibodies, after subsequent transfer, no anti-NDH-reactive subunit was detected when compared with the positive control with thylakoid extract from *H. vulgare*. However, NDH-F and NDH-J antibodies do recognize the corresponding polypeptides when the *T. plicata* extract was subjected to denaturing SDS-PAGE (right side of Fig. 2). Based on their migration, the presumptive NDH-F and NDH-J polypeptides of *T. plicata* have slightly lower and slightly higher, respectively, sizes than their homologous *H. vulgare* polypeptides (70 and 20 kDa). Several causes could explain the failure to detect NDH polypeptides of *T. plicata* after native electrophoresis. One possibility is that the Ndh complex of *T. plicata* could be highly unstable and collapses during thylakoid solubilization with 2% Triton X-100. Hence, disassembled subunits would migrate out of the gel during native electrophoresis. Alternatively, the Ndh complex could be so tightly bound to thylakoids that it can not be solubilized with 2% Triton X-100, whereas its subunits could be solubilized by boiling in the presence of SDS. Therefore, *T. plicata*, as well as the other four tree species, probably contains an active Ndh complex.

### Post-illumination fluorescence indicative of Ndh complexes

Amino acid sequence variations of NDH subunits among different plants must affect both the recognition by each antibody raised against the *H. vulgare* subunits and the nitroblue tetrazolium: NADH oxido-reductase activity of the Ndh complex. In addition, the activity (as detected in zymograms) of Triton X-100 extracts is rapidly lost, which makes the comparison of relative activities in different plants as well as of the level of activity during the different physiological stages of the leaves difficult. Post-transcriptional control of *ndh* gene expression (Del Campo et al. 2000, Del Campo et al. 2002, Serrot et al. 2008) and post-translational modification of the *ndh* gene products (Lascano et al. 2003, Martín et al. 2009) also reduce the relevance of northern and western assays to assess changes of the Ndh complex activity in vivo. The above-mentioned limitations as well as the uncertainty regarding the presence of active Ndh complex in *T. plicata* prompted us to investigate the chlorophyll fluorescence increase after relative high to minimum light transition (post-illumination fluorescence) in the five trees. This assay is commonly accepted (Burrows et al. 1998, Kofer et al. 1998, Shikanai et al. 1998, Martín et al. 2004, 2009, Wang and Portis 2007) as a valid test of the plastoquinone reduction by the Ndh complex in vivo.

**Fig. 3:**
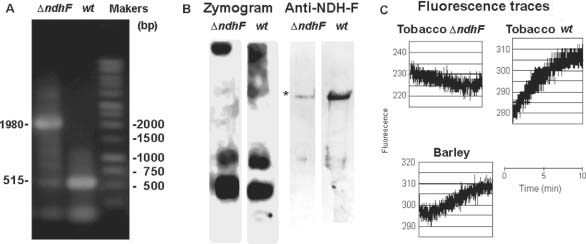
Controls relating post-illumination chlorophyll fluorescence to the Ndh complex. (A) PCR amplification products using primers F2/F4 for the *ndhF* gene sequence and DNA isolated from Δ*ndhF* and *wt* tobaccos. Sizes of the main amplified fragments and of some markers are indicated in the left and right, respectively. (B)Solubilized thylakoid proteins from Δ*ndhF* and *wt* tobaccos were separated by native electrophoresis and revealed for NADH dehydrogenase activity (Zymogram) and, after membrane blotting, immunoassayed with antibody against the NDH-F polypeptide (anti-NDH-F lanes). (C) Chlorophyll fluorescence traces after relative high to minimum light transition. Assays were performed with leaves of tobacco (Δ*ndhF* and *wt*) and barley as described in section Materials and methods. The traces shown are only those of fluorescence readings every 0.1 s during the 9 min following the final 0.15–0.1 µmol photon m^−2^ s^−1^ PAR transition. Vertical axes show the relative fluorescence readings.

Fig. 3 shows controls relating post-illumination fluorescence to the presence of active thylakoid Ndh complex in barley and tobacco. The Ndh defective tobacco (Δ*ndhF*) was obtained (Martín et al. 2004) by insertion, between nucleotide positions 1023 and 1024 of the *ndhF* reading frame, of a 1465-bp construction containing appropriate promoters and transcription terminators flanking the spectinomycin resistance gene (*aadA*). When PCR-amplified with the primer pair F2/F4 (flanking the insertion position), DNAs of Δ*ndhF* and *wt* tobaccos show the predicted 1980 and 515 bp, respectively, as the main amplified bands (Fig. 3A). The presence of a faint 515 band in the Δ*ndhF* lane indicates that more than six generations under spectinomycin selection had not yet produced homoplastomic Δ*ndhF* tobacco. With most its *ndhF* gene copies essentially disrupted, Ndh activity and anti-NDH-F reactive bands were almost undetectable in Δ*ndhF* when compared to *wt* (asterisk in Fig. 3B) and, as Fig. 3C shows, the post-illumination fluorescence increase of *wt* tobacco became a slight fluorescence decrease in Δ*ndhF* tobacco. In accordance with the Ndh activity of barley shown in Fig. 2, Fig. 3C also shows that barley leaves exhibit the characteristic post-illumination chlorophyll fluorescence increase.

**Fig. 4:**
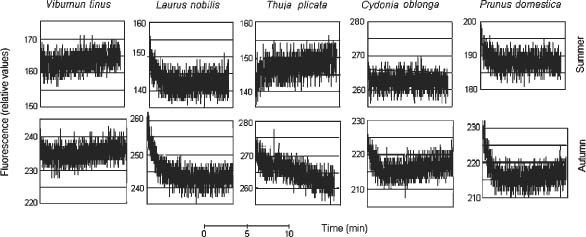
Chlorophyll fluorescence traces after relative high to minimum light transition. Assays were performed as described in section Materials and methods with leaves of the indicated trees in summer (June and July) and autumn (October and November). The traces shown are only those of fluorescence readings every 0.1 s during the 9 min following the final 0.15 to 0.1 µmol photon m^−2^ s^−1^ PAR transition. Vertical axes show the relative fluorescence readings.

Leaf fluorescence assays in tree leaves were carried out in situ in summer (June to July) and autumn (October to November) and repeated three to nine times. The results obtained did not differ significantly for the same plant and condition. As Fig. 4 shows, fluorescence increased in *V. tinus* and *T. plicata* in summer assays after transition to minimum light. During the same months, the fluorescence increase under minimum light was barely detectable in *L. nobilis*, *C. oblonga* and *P. domestica*, sometimes after an initial decline. The fluorescence response significantly changed in autumn in *C. oblonga* and *P. domestica*, in which it increased more rapidly than in summer, and in *T. plicata* in which the autumnal fluorescence decrease contrasted with the increase in summer. On the bases of results by different groups mentioned above and those in Fig. 3 indicating a close correlation between presence of active Ndh complex and increase of post-illumination fluorescence, the results shown in Fig. 4 strongly indicate that the leaves of the five trees assayed contain the functional Ndh complex transferring electrons to plastoquinone. The autumnal increase in the post-illumination fluorescence in *C. oblonga* and *P. domestica*, both deciduous trees, strongly contrasts with the lower (*L. nobilis*), unchanging (*V. tinus*) or decreasing (*T. plicata*) fluorescence in evergreen trees.

### *ndh* genes in *T. plicata*: sequence of the *ndhB* gene

With the exception of non-photosynthetic parasitic plants and recent species of the *Erodium* genera (Blazier et al. 2011), all angiosperms tested contain the plastid *ndh* genes (Martín and Sabater 2010). However, the plastid genomes of certain gymnosperm investigated (mainly Gnetales and Pinaceae) lack *ndh* genes (Wakasugi et al. 1994, McCoy et al. 2008, Braukmann et al. 2009, Martín and Sabater 2010). As no *ndh* gene has yet been reported in *T. plicata* to complement western and fluorescence evidences for the presence of the Ndh complex in this gymnosperm, we looked for evidences of *ndh* genes and completely sequenced the *ndhB* gene and its transcript.

**Fig. 5:**
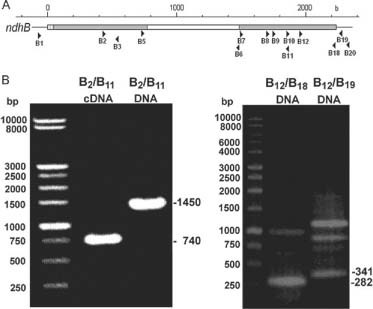
Map and amplification products of the *ndhB* gene. (A) Scale map of the *ndhB* gene showing the two exons (gray boxes), the intron (white box) and the position and orientation of the primers used in this work. The dotted small box corresponds to the 5′-end sequence present in the angiosperm gene but not in the gymnosperm gene whose start codon is 18 codons downstream with respect to that of angiosperms. (B) Agarose gel electrophoresis of PCR and RT-PCR amplification products obtained with B_2_/B_11_ primer pair. DNA and RNA of *Thuja plicata* were, respectively, PCR (DNA lanes) and RT-PCR (cDNA lane) amplified. (C) DNA amplification with primer pairs B_12_/B_18_ and B_12_/B_19_.

For this purpose, we analyzed the PCR amplification products obtained from DNA and RNA preparations of *T. plicata* with different primer pairs of known angiosperm *ndhB* sequences and of partial sequences (see below) obtained successively in *T. plicata*. In most plants, the plastid *ndhB* gene contains an intron of around 710 bp, which separates exon 1 (around 775 bp) from exon 2 (around 755 bp). Fig. 5A shows the positions and orientation of the primers used in this investigation on the map that was deduced of the *ndhB* gene of *T. plicata*. By selecting appropriate combinations of primers, we amplified overlapping fragments covering the entire *ndhB* gene. Primary and nested amplifications from plant DNA and extensive sequencing of the amplification products obtained with primer pairs B_1_/B_3_, B_2_/B_11_, B_5_/B_6_, B_7_/B_19_, B_9_/B_19_, B_10_/B_19_ and B_12_/B_19_ confirmed the co-linearity of sequences and the presence in *T. plicata* of the two exons and intron of the *ndhB* gene. cDNA was also sequenced after reverse transcriptase PCR (RT-PCR) amplification. As examples, Fig. 5B shows that the B_2_/B_11_ primer pair amplified a 1450-bp fragment from the genomic DNA template including the *ndhB* intron (lane DNA), whereas the same primer pair amplified an approximately 740-bp fragment from the cDNA template that lacked the intron (cDNA lane of pair B_2_/B_11_).

Subfragments of 300–400 bp were sequenced at least twice from the fragments amplified with primer pairs B_1_/B_3_, B_2_/B_11_, B_5_/B_6_, B_7_/B_19_, B_9_/B_19_, B_10_/B_19_, B_12_/B_19_, B_8_/B_18_, B_12_/B_18_, B_7_/B_20_, B_9_/B_20_ with both DNA and cDNA of *T. plicata*. The results consistently revealed a single *ndhB* sequence for the exon 1 (723 bp), the intron (708 bp) and the first 406 bp of the exon 2. However, two different sequences were found for the remaining 350 bp of the exon 2. One 3′ end 350 bp sequence (sequence 1) was obtained when B_19_ was used as 3′end primer and a second sequence (sequence 2) was obtained in fragments amplified with B_18_ or B_20_ as the 3′ end primer, both in DNA as well as in cDNA. The right side of Fig. 5B shows the 282-bp fragment amplified with the B_12_/B_18_ primer pair and the several fragments amplified with the B_12_/B_19_ primer pair of which only the 341-bp marked fragment had the 3′ end sequence of the *ndhB* gene. Regardless of the 3′ end primer, all sequenced fragments of the *ndhB* gene have the same 5′ end sequence up to 406 bp of the exon 2. We conclude that *T. plicata* has, at least, two different *ndhB* genes: *ndhB*1 (Accession No. EF421240) and *ndhB*_2_ (Accession Nr. EF421241) that differ only in the last 350 bp, and that both genes are transcribed. In both genes, exon 1, the intron and approximately the first half sequence of exon 2 are identical.

**Fig. 6:**
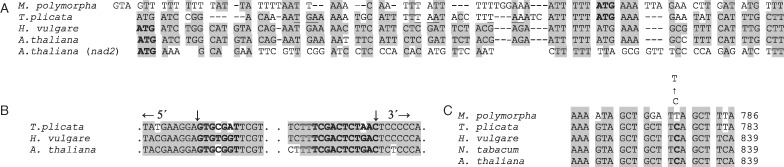
Eye alignments of selected sequences of *ndhB* genes and *nad2* gene. All sequences are of the plastid *ndhB* gene except, when indicated, those of the mitochondrial *nad2* used for comparison. Bases conserved with respect to the *Hordeum vulgare ndhB* reference sequence are grey shadowed. (A) 5′-ends of plastid *ndhB* genes of *Marchantia polymorpha*, *Thuja plicata*, *H. vulgare* and *Arabidopsis thaliana* mitochondrial *nad2* gene, showing the different location of the start codon (in bold, ATG). Underlined bases in *T. plicata* are stop codons. (B) Characteristic 5′- and 3′-ends of the intron indicating the consensus bases of the chloroplastic group II intron in bold and the cleavage sites (↓). (C) Sequences around the C (in bold) that is edited to U in the *ndhB* gene transcripts of higher plants. The numbers of the last bases correspond to those in mature transcripts lacking the intron.

Sequence alignments of the *ndhB* genes (Fig. 6A) show that the ATG start codon of the *T. plicata ndh B* gene is located 18 codons downstream from that of angiosperms and of the homologous *nad2* gene of *Arabidopsis thaliana* encoding the corresponding polypeptide of the mitochondrial complex I. The initiation of translation in the *ndhB* gene of *T. plicata* coincides with the ATG start codon of the gene of the liverwort *Marchantia polymorpha* and other gymnosperms (not shown). Several in-phase stop codons, as well as the deletion and insertion of a few bases clearly indicate that the first ATG (shown in *T. plicata* but not present in *M. polymorpha*) can not function as a start codon of the *ndhB* gene of *T. plicata*. These results indicate that, in the transition from gymnosperms to angiosperms, the *ndhB* gene was enlarged by 54 bp. The boundaries of the *ndhB* intron in *T. plicata* show the characteristic sequences of the chloroplastic type II introns: GTGC(T)GA(G)T…TCGACTCTA(G)AC (Fig. 6B). When comparing with cDNA sequences, we found only one codon that undergoes C to U editing of the primary transcript in *T. plicata*: codon 259 (TCA encoding Ser is edited to TTA encoding Leu, Fig. 6C). The homologous codon 277 is also edited in the *ndhB* transcripts of the angiosperms *H. vulgare*, *N. tabacum* and *A. thaliana* (Freyer et al. 1995, Tillich et al. 2005; Fig. 6C).

To further support evidence of the presence of the Ndh complex, we also PCR amplified and sequenced regions of other plastid *ndh* genes of *T. plicata* (not shown): the *ndhC* (completely) and *ndhK* (partially) genes (Accession Nos. EF421243 and EF421244, respectively).

## Discussion

The low amounts and instability of the Ndh complex poses a formidable challenge to the investigation of the functional role of the Ndh complex. On the other hand, the low expression levels and the complex post-transcriptional processing of the primary transcripts of plastid *ndh* genes make the investigation of the factors that control the Ndh complex at the genetic level appealing, but one must proceed with caution. At present, antibodies raised against subunits of the Ndh complex of rapid-growth monocarpic plants provide a valuable tool in the research for homologous subunits in still unexplored plants such as trees, especially when combined with zymographic approaches. However, as demonstrated by our results with *T. plicata*, the failure to immunodetect Ndh subunits after native electrophoresis does not exclude the presence of the Ndh complex. The characterization of the Ndh complex requires its purification, as accomplished in a few monocarpic plants, but preliminary, necessary and complementary approaches include the comparison of zymographic, immunodetection and fluorescent assays. In addition, the presence of *ndh* genes in one plant strongly suggests (but does not prove) the presence of the Ndh complex. Therefore, the investigation of the functional role of Ndh complex and *ndh* genes in trees should require, at least, the three experimental approaches tested here.

The immunoblot bands in tree preparations (Fig. 1) were not as sharp as in barley and tobacco (Figs 1 and 2) against whose NDH subunits the antibodies were raised (Martín et al. 2009).These facts and minor lane distortions during electrophoretic migration and transfer from gel to membrane make it sometimes difficult to accurately identify the zymographic band corresponding to the Ndh complex in trees. However, the presence of NADH dehydrogenase bands co-migrating in native electrophoresis with a complex containing the NDH subunits strongly indicates that NADH is electron donor in the Ndh complexes of the trees assayed, as well as in barley (Cuello et al. 1995, Casano et al. 2000), peas (Sazanov et al. 1998), potato (Corneille et al. 1998), brassica (Díaz et al. 2007) and tobacco (Martín et al. 2004, 2009). When mutant plants are available, as in Fig. 3 here, the reported NADH-dependent activities were impaired in control mutant plants deficient in at least one plastid *ndh* gene.

It is generally accepted that plastoquinone is the electron acceptor of the Ndh complex reaction; however, there is disagreement regarding the electron donor. Persistent difficulties involved in finding the NADH-binding subunit of the Ndh complex have prompted proposals of alternative electron donors. Yamamoto et al. (2011) assayed the Ndh activity in preparations of washed *Arabidopsis* thylakoids by recording the post-illumination increase of chlorophyll fluorescence in the presence antimycin A, which inhibits the main (if not the only) cyclic electron transport chain (Kurisu et al. 2003). These results lead Yamamoto et al. (2011) to conclude that ferredoxin is the electron donor to the Ndh complex. Control assays with nuclear mutants of *Arabidopsis* that affect the Ndh activity suggest that a nuclear encoded protein (CRR31), that accumulated in thylakoids independently of the Ndh complex (Yamamoto et al. 2011), could provide a link between ferredoxin and the Ndh complex. Differences in the assays of the activity and the widely accepted instability of the Ndh complex, which could affect the integrity of the complex, could be involved in the disagreement regarding the electron donor that, therefore, should be further investigated.

After zymographic, immunological and molecular evidences and on the bases of the close correlation between the presence of *ndh* genes and the increase of post-illumination chlorophyll fluorescence (Burrows et al. 1998, Kofer et al. 1998, Shikanai et al. 1998, Martín et al. 2004, 2009, Wang and Portis 2007; Fig. 3), the increases of chlorophyll fluorescence in at least one season in most tree leaves assayed seem the best indication of the presence of functional Ndh complexes. Although the assays of chlorophyll fluorescence must not linearly reflect the rate of plastoquinone reduction, seasonal differences in the rates of fluorescence increase in one plant plausibly reflect differences of the in vivo activity of the Ndh complex in that plant and suggest that, when properly calibrated for each species, the rate and shape of the post-illumination chlorophyll fluorescence increase could provide a rapid field test for monitoring changes of the in vivo Ndh activity during the year and under different environmental conditions. In this regard, perennial plants could provide an excellent model to assess the functional role of the Ndh complex. The increased autumnal fluorescence in deciduous plants (Fig. 4) could suggest that a higher activity of the thylakoid Ndh complex is associated to leaf senescence as demonstrated in tobacco (Zapata et al. 2005) and suggested in other systems (Reape et al. 2008, Huang and Braun 2010, Nilo et al. 2010).

The plastid DNA of angiosperms contains two *ndhB* genes that map in the inverted repeated region and, therefore, have exactly the same sequence. At first sight, the presence of two *ndhB* genes in *T. plicata* differing in the 350-bp 3′ end sequences seems surprising. One possible explanation could be that the inverted repeated region of the *T. plicata* plastid DNA is shorter than that of angiosperms and, consequently, the 350-bp 3′ ends of the two *ndhB* genes extend into the large single copy region. Hence, the two 350-bp 3′ ends have evolved independently accumulating different base changes. In this regard, it is worthy to note a partially similar condition of the short end regions of the *ndhH* and *ndhF* genes that map in the small single copy region of plastid DNA, but extend into the inverted repeated regions (and, therefore, are repeated) of, respectively, maize and rice plastid DNA (Maier et al. 1995).

**Fig. 7:**
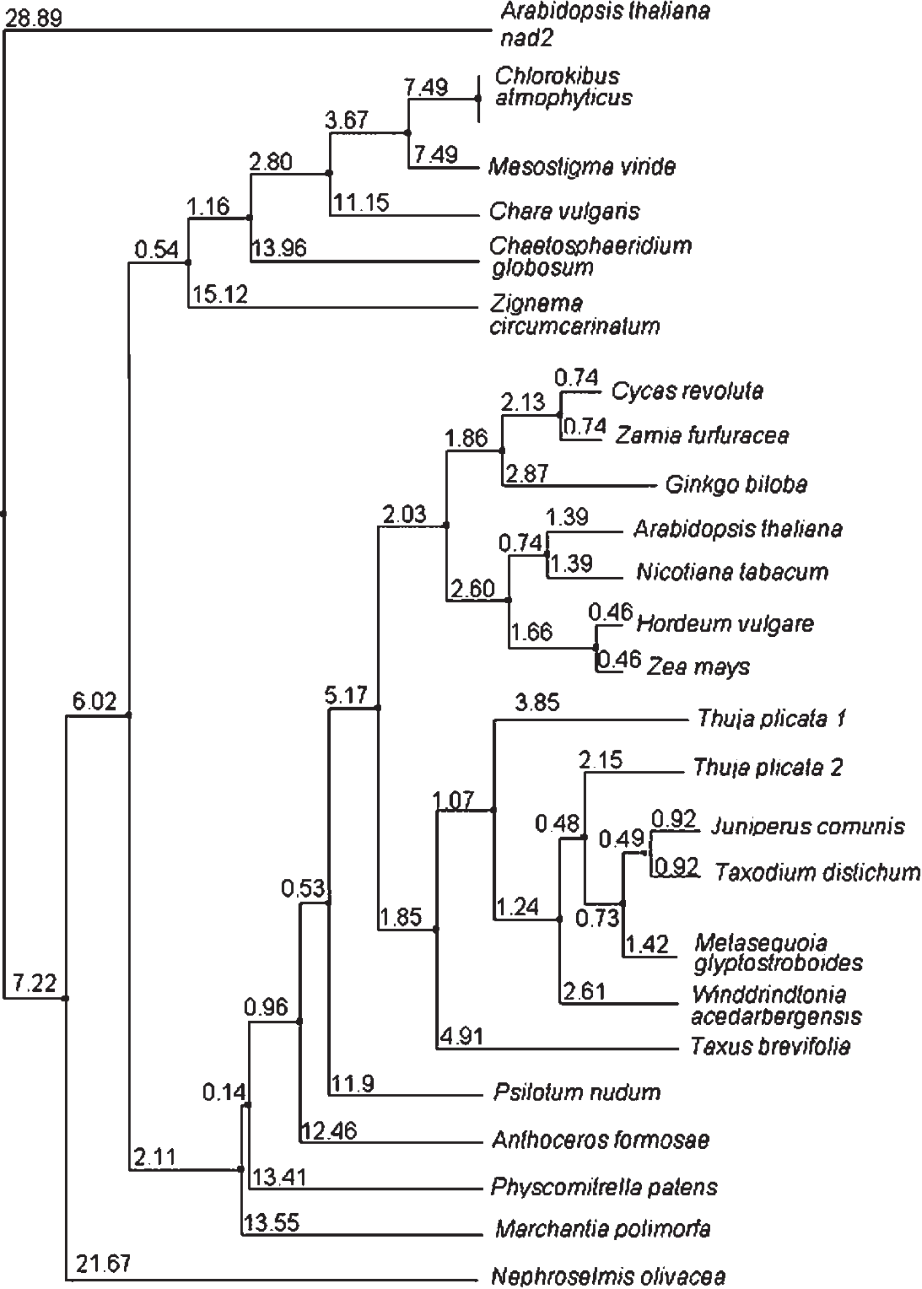
Phylogenetic relationships among different plants based on the amino acid sequence of the *ndhB* gene protein deduced from base sequences in Data Bank. The average distance tree (inserted numbers) was constructed as described in section Materials and methods using the PID. For a comparison, the homologous protein of the mitochondrial *nad2* gene of *Arabidopsis thaliana* is also included. The genes of *Thuja plicata* 1 and *T. plicata* 2 are those reported in this publication.

Phylogenetic analysis demonstrates that the two sequences reported here correspond to plastidial *ndhB* and not mitochondrial *nad2*. The well-known significant sequence dissimilarity between mitochondrial *nad* and plastidial *ndh* genes is shown in the phylogenetic tree of Fig. 7, constructed from the protein products of the *ndhB* genes of several photosynthetic organisms and the homologous mitochondrial *nad2* gene of *A. thaliana*. As expected, the common root for *nad2* and *ndhB* is at the ancestor of algae and the sequences reported here for *T. plicata ndhB* genes group in a gymnosperm branch whose nearest branch is that of angiosperms. The large distance between mitochondrial *nad* and plastidial *ndh* genes explains why antibodies do not usually cross-react with mitochondrial and plastid polypeptides(Guéra et al. 2000). The complete sequences described here for the *ndhB* genes of *T. plicata* show homology with several partial sequences described in gymnosperms. It is noteworthy that the gymnosperms *Cycas revoluta*, *Zamia furfuracea* and *Ginkgo biloba* group together in a branch closer to angiosperms than that including the other gymnosperms, which agrees with the phylogeny analysis based on concatenated plastid protein genes (Wu et al. 2007). A more extensive phylogenetic analysis based on the available 80 *ndhB* sequences closest to that of *T. plicata* (Appendix S1, supporting information), indicates several distinguishable gymnosperm branches and situated *Thuja* with *Metasequoia*, *Cryptomeria*, *Taxodium*, *Juniperus* and *Widdringtonia* in the Cupresaceae branch.

## Abbreviations

RT-PCR: reverse transcriptase polymerase chain reaction
PID: percentage identity

## Supporting Information

**Appendix S1.** Phylogenetic relationships based on theplastid *ndhB* gene.
