# Supplementary material for: Activity, polypeptide and gene identification of thylakoid Ndh complex in trees: potential physiological relevance of fluorescence assays
Source: Physiol Plant. 2012 Sep;146(1):110–20. doi: 10.1111/j.1399-3054.2012.01598.x (PMC3457125; doi:10.1111/j.1399-3054.2012.01598.x)

Appendix S1.

Phylogenetic relationships among different plants based on the 80 sequences in Data Bank closest to the *ndhB* gene (*ndhB1*) of *Thuja plicata*. The relative distance tree was constructed via the web at the NCBI server using the Fast Minimum Evolution method. Gymnosperm branches are expanded to show relationships among species that situate *T. plicata* within the Cupresaceae (lower box). Upper box includes angiosperms.


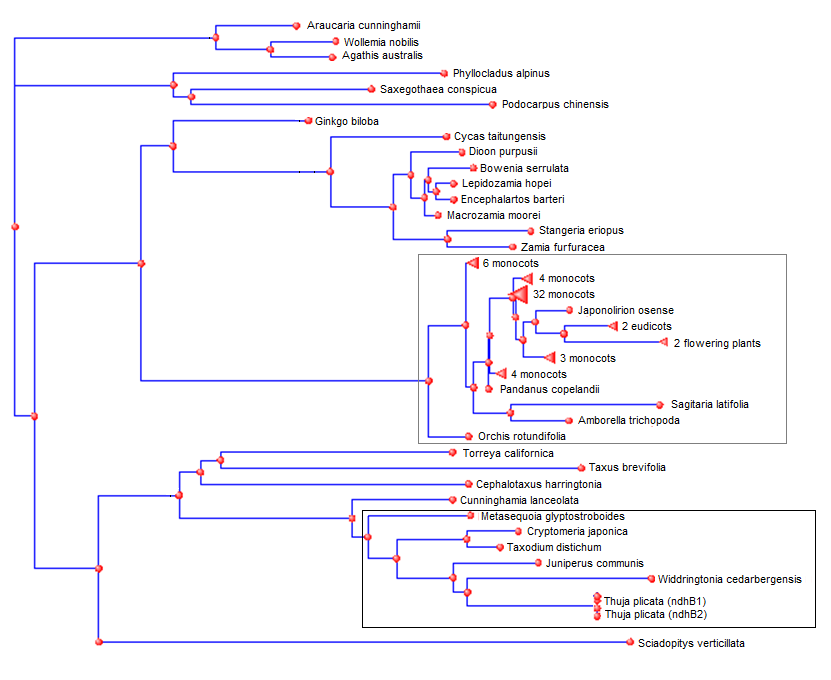

Supplement: Supplementary file 1 [file ppl0146-0110-SD1.doc]
